# Characterization of a barley (*Hordeum vulgare* L.) mutant with multiple stem nodes and spikes and dwarf (*msnsd*) and fine-mapping of its causal gene

**DOI:** 10.3389/fpls.2023.1189743

**Published:** 2023-07-06

**Authors:** Dandan D. Qin, Rui Liu, Fuchao Xu, Guoqing Dong, Qing Xu, Yanchun Peng, Le Xu, Hongna Cheng, Ganggang Guo, Jing Dong, Chengdao Li

**Affiliations:** ^1^ Institute of Food Crops, Hubei Academy of Agricultural Sciences, Hubei, Wuhan, China; ^2^ Hubei Key Laboratory of Food Crop Germplasm and Genetic Improvement, Hubei, Wuhan, China; ^3^ Key Laboratory of Crop Molecular Breeding, Ministry of Agriculture and Rural Affairs, Hubei, Wuhan, China; ^4^ School of Life Science and Technology, Wuhan Polytechnic University, Hubei, Wuhan, China; ^5^ Ministry of Agriculture and Rural Affairs (MARA) Key Laboratory of Sustainable Crop Production in the Middle Reaches of the Yangtze River (Co-construction by Ministry and Province), College of Agriculture, Yangtze University, Hubei, Jingzhou, China; ^6^ Institute of Crop Sciences, Chinese Academy of Agricultural Sciences, Beijing, China; ^7^ Western Crop Genetics Alliance, College of Science, Health, Engineering and Education, Murdoch University, Perth, WA, Australia

**Keywords:** barley, BSR-seq, TCP, multiple stem nodes and spikes and dwarf, fine-mapping

## Abstract

**Introduction:**

Multiple nodes and dwarf mutants in barley are a valuable resource for identifying genes that control shoot branching, vegetative growth and development.

**Methods:**

In this study, physiological, microscopic and genetic analysis were conducted to characterize and fine-map the underling gene of a barley mutant with Multiple Stem Nodes and Spikes and Dwarf (*msnsd*), which was selected from EMS- and ^60^Co-treated barley *cv*. Edamai 934.

**Results and discussion:**

The *msnsd* mutant had more stem nodes, lower plant height and a shorter plastochron than Edamai 934. Moreover, the mutant had two or more spikes on each tiller. Microscopic analysis showed that the dwarf phenotype of *msnsd* resulted from reduced cell lengths and cell numbers in the stem. Further physiological analysis showed that *msnsd* was GA^3^-deficient, with its plant height increasing after external GA^3^ application. Genetic analysis revealed that a single recessive nuclear gene, namely, Hv*MSNSD*, controlled the *msnsd* phenotype. Using a segregating population derived from Harrington and the *msnsd* mutant, Hv*MSNSD* was fine-mapped on chromosome 5H in a 200 kb interval using bulked segregant analysis (BSA) coupled with RNA-sequencing (BSR-seq), with a C-T substitution in the exon of HvTCP25 co-segregating with the *msnsd* phenotype. RNA-seq analysis showed that a gene encoding gibberellin 2-oxidase 8, a negative regulator of GA biosynthesis, was upregulated in the *msnsd* mutant. Several known genes related to inflorescence development that were also upregulated and enriched in the *msnsd* mutant. Collectively, we propose that Hv*MSNSD* regulates the plastochron and morphology of reproductive organs, likely by coordinating GA homeostasis and changed expression of floral development related genes in barley. This study offers valuable insights into the molecular regulation of barley plant architecture and inflorescence development.

## 1 Introduction

Globally, barley (*Hordeum vulgare* L.) is the second most important temperate cereal crop after wheat. Shoot architecture is a major determinant of the function, diversification and adaptation of barley. It is largely defined by plant height, leaf arrangement, branching patterns and inflorescence morphology (Wang et al., 2018). These features are determined by the activity and fate of the shoot apical meristem, axillary meristems (AXMs), leaf meristems and intercalary meristems (McKim, 2019). Typically, a single AXM initiates and forms in each leaf axil, which together with the leaf, node and subtending internode, generates a phytomer unit (Mcmaster, 2005). Plastochron refers to the time interval between the formation of two successive leaves, representing the temporal pattern of leaf initiation. Plastochron and phyllotaxy are two major contributors to plant architecture (Hibara et al., 2021). In rice (*Oryza sativa* L.), three genes responsible for regulating rapid leaf development—*PLASTOCHRON 1* (*PLA1*) (Miyoshi et al., 2004), *PLA2* (Kawakatsu et al., 2006) and *PLA3* (Kawakatsu et al., 2009)—have been identified, encoding a plant-specific cytochrome P450 protein family, the MEI2-like RNA-binding protein and glutamate carboxypeptidase, respectively.

Barley *multiple nodes and dwarf* (*mnd*) mutants have more stem nodes but significantly lower plant heights than wild-type plants. Several *mnd* mutants, such as 93–597 derived from barley line ‘6121’ (Zhang and Zhai, 1995) and 76–2104 derived from ‘Zaoshu 3’ (Kawakatsu et al., 2006), have been reported since 1922 (Harlan and Pope, 1922). Apart from abnormal stem structure, the *mnd* mutations always exert pleiotropic effects on other agronomic traits, producing shorter leaf blades and aberrant inflorescences. Consistent with the increased stem node and leaf numbers, a shorter plastochron is usually observed in these mutants (Mascher et al., 2014). For these mutants, three underlying genes have been identified in barley—*MND1*, *MND4* and *MND8*. *MND4* is a member of the CYP78A family of cytochrome P450 enzymes, an ortholog of the rice *PLA1* gene. Variations in *MND4* were associated with the “multiple nodes but dwarf” phenotypes in barley, such as SNPs, which change the encoded amino acids, introducing premature stop codons, disrupting the splice site and partially or completely deleting this gene (Mascher et al., 2014). *MND1* and *MND8* were identified recently. *MND1* located on chromosome 7H and encoded an Acyl-CoA N-acetyltransferase-like protein (Walla et al., 2020), while *MND8* located on chromosome 5H and encoded a MATE transporter-family protein (Hibara et al., 2021).

Spike morphology is another determining factor for grain yield in the grass family. Barley inflorescence architecture differs from other Poaceae species, such as rice, wheat and maize. Barley specifically develops a branchless, spike-shaped inflorescence, where grain-producing spikelets are attached directly to the main axis (Gauley and Boden, 2019; Koppolu and Schnurbusch, 2019). Two-row and six-row differentiation is specific to barley spikes, with *Vrs1* (Komatsuda et al., 2007), *Vrs2* (Youssef et al., 2017), *Vrs3* (Bull et al., 2017), *Vrs4* (Koppolu et al., 2013) and *Vrs5* (Ramsay et al., 2011) being the known row-type genes in barley. Besides row-type, branched spike is another variation observed in barley spike morphology. The grass-specific Teosinte branched 1/Cycloidea/Proliferating cell factor (TCP) family genes that encode transcription factors, such as the CYC/TB1-type *TCP* (Shang et al., 2020) and *COMPOSITUM1* (Poursarebani et al., 2020), play a crucial role in branched spike formation in barley. Moreover, loss-of-function of the barley SEPALLATA MADS-box protein *HvMADS1* is responsible for maintaining the branched inflorescence-like structure at high ambient temperatures (Li et al., 2021). In addition to these genes, a novel branched-spike gene in barley has been mapped on chromosome 2H in a 5 cM interval (Wang et al., 2020), with major and minor QTLs also identified for lateral spikelet indeterminacy and supernumerary spikelet phenotype in the central spikelets based on the genetic analysis of the *multiflorus2.b* mutant (Koppolu et al., 2022). However, for most *mnd* mutants, more than one spike per tiller, especially on the main tiller, can be produced, significantly different from the typical branched spike and potentially beneficial for improving barley yield.

A barley mutant with multiple stem nodes, spikes and dwarf (*msnsd*) was identified from chemically and physically treated cv. Edamai 934 (E934 hereafter) (Qin et al., 2021), an elite feed barley variety developed by Hubei Academy of Agricultural Sciences. Here, we undertook phenotypic and genetic characterization of *msnsd* and its causal gene *HvMSNSD* using BSR-seq. This study will not only enrich our understanding of barley plant architecture and inflorescence development, but also provide valuable resources for barley dwarf and yield breeding.

## 2 Materials and methods

### 2.1 Comparison of agronomic traits of *msnsd* and its parent E934

The *msnsd* mutant, characterized with “Multiple Stem Nodes, Spikes and Dwarf”, was generated from ethyl methane sulfonate (EMS)- and ^60^Co-treated feed barley variety E934 (Qin et al., 2021). The *msnsd* mutant and E934 were grown in the field at the experimental station of Hubei Academy of Agricultural Sciences, Wuhan, China, with management practices following the local standard practices. At maturity, plant height (PH), node number on the main tiller (NN), number of spikes per plant (NSP), length of the main spike (LS) and grain number on the main spike (GNS) were measured on ten plants. The average values for each parameter were used to determine differences between *msnsd* and E934. All tillers were removed at the five-leaf stage to determine the plastochrons of *msnsd* and E934. The fifth leaf on the main tiller was labeled, with leaf number recorded weekly.

### 2.2 Microscopic analysis of stem cells and immature spikes of *msnsd* and E934

Stem sections in the center of *msnsd* and E934 plants at the heading stage were sliced lengthwise into 10 mm sections and fixed in 10% formalin solution for 48 h at room temperature. Next, the tissue was dehydrated in a series of graded ethanol solutions to displace the water and then infiltrated with wax. The infiltrated tissues were then embedded into wax blocks and placed in the microtome for sectioning. Thereafter, sections were deparaffinized and rehydrated with distilled water. The slides were immersed in Safranin O solution for 1–2 h, rinsed in tap water to remove excess dye and dehydrated successively with a graded series of alcohol (50%, 70% and 80%), each for 3–8 s. The slides were then immersed in the fast green solution for 30–60 s and dehydrated in absolute alcohol three times. Finally, they were cleared in xylene for 5 min and mounted with the resin mounting medium. Cells of *msnsd* and E934 were observed in a bright field using an Olympus BX51 photomicroscope. All these procedures were carried out at the Wuhan Servicebio Technology Co., Ltd (Wuhan, China). Transverse sections of the stem were checked following the same procedure.

Immature spikes from *msnsd* and E934 plants at the booting stage were pre-fixed with 2.5% glutaraldehyde in a phosphate buffer (pH 7.0) at 4°C overnight. Subsequent treatments of the samples were conducted at the Wuhan Servicebio Technology Co., Ltd, which were then observed by scanning electron microscope (Hitachi TM-100) according to the manufacturer’s instructions (Qin et al., 2015).

### 2.3 Hormone response test

The *msnsd* and E934 plants at the five-leaf stage on Jan 13, 2021, were tested for their responses to five phytohormones, including 50 mg/L gibberellic acid 3 (GA_3_), 200 µM 3-indoleacetic acid (IAA), 50 µM 6-benzyl aminopurine (6-BA), 50 µM 1-aminocyclopropane-1-carboxylic acid (ACC) and 100 µM ABA. Around 15 mL of each phytohormone was poured directly into the surrounding soil, with water-treated seedlings serving as the control. The *msnsd* and E934 plants were sprayed with 50 mg/L GA_3_ and Tween 20 after the appearance of spikes. All treatments were applied to five plants of similar seedling length. After 20 days of treatment, plant height of the treated and control plants was recorded weekly until maturity. Agronomic traits, including tiller number, stem node number, internode length on the main tiller, spike number on the main tiller, leaf number and spikelet number per spike were determined after harvest.

### 2.4 Quantification of endogenous GAs

E934 and *msnsd* were grown in the field under normal conditions in autumn 2020. At the booting stage, GA contents (GA_1_, GA_3_, GA_4_, GA_7_ and GA_20_) in the young apical meristem (excluding inflorescence) of *msnsd* and E934 were determined using LC-MS/MS (Mubarak et al., 2012) at the Wuhan Greensword Creation Technology Co. Ltd. Three independent biological replicates and three technical replicates were measured for each sample.

### 2.5 Genetic analysis of the gene controlling the *msnsd* phenotype in barley

Genetic characterization of the gene (s) controlling the *msnsd* phenotype used the phenotypes of the F_1_ hybrid, F_2_ population and F_2:3_ family lines derived from Harrington and *msnsd* plants. Harrington is a two-row, malting barley variety from Canada. Chi-squared test was used to determine the suitability of observed data with the expected segregation ratios.

### 2.6 BSR-sequencing analysis based on BSA and RNA-sequencing

To map the causal gene for *msnsd*, bulked segregant RNA-seq (BSR-seq) based on bulked segregant analysis (BSA) and RNA-sequencing (RNA-seq) was conducted in two pools with distinct phenotypes from the F_2_ population. Five seeds from 40 wild-type and 40 mutants with multiple stem nodes and multiple spikes from F_2_ individuals were sown and grown at 25°C for two weeks to extract high-quality RNA. A half centimeter’s leaves from all the 200 wild-type or mutant seedlings were pooled and collected to construct wild-type or mutant bulks for RNA isolation. RNA was extracted following the TRIzol protocol (Rio et al., 2010). RNA-seq was undertaken using the HiSeq 2500 platform (Illumina) following the manufacturer’s protocol. RNA extraction and RNA-seq were conducted at the Beijing Novogene Bioinformatics Technology Co. Ltd. Implementation of single nucleotide polymorphism (SNP) calling and the filtration and identification of SNPs and Indels associated with the *msnsd* phenotype were performed as described elsewhere (Wang et al., 2017; Zhang et al., 2020b). High-quality reads were aligned to the barley Morex-V1 assembly. Thereafter, the mapping results were filtered using in-house Perl Scripts. Only uniquely mapped reads with a Phred quality value > 40 were kept.

### 2.7 Validation of the BSR-seq results and preliminary mapping of *HvMSNSD*

Seedling DNA from the F_3_ progeny of 46 wild-type (24 individuals were homozygous and 22 were heterozygous according to performance of F_2:3_) and 37 mutants of F_2_ individuals was extracted using CTAB methods to validate the BSR-seq analysis. Indel markers in the target region were developed according to the BSR-seq analysis and published results (Zhou et al., 2015). These markers were then applied to the two parents, separated on 8% non-denaturing polyacrylamide gels and visualized after silver staining. Subsequently, polymorphic markers were used to genotype the resulting sub-pools of F_3_ (each derived from one F_2_ individual) to validate the BSR-seq data and preliminarily map the candidate gene.

### 2.8 Fine-mapping of *HvMSNSD*

Indel markers linked to *HvMSNSD* were applied to the F_2_ population comprising of 460 progenies to fine-map the gene. The phenotype of F_2_ individuals was confirmed by evaluating their own and F_2:3_ performance under field conditions. Several SNP markers in the target region were further developed based on the BSR-seq analysis and applied to the recombinant lines using the Penta-primer amplification refractory mutation system (PARMS) at Gentides Biotech Co., Ltd, Wuhan, China. Furthermore, 1,869 F_2:3_ and F_3:4_ homozygous mutant individuals derived from the heterozygous F_2_ and F_3_ lines were used to confirm and fine-map the candidate gene.

### 2.9 Cloning and analysis of candidate genes

High-confidence genes in the target region were identified according to the annotated barley reference genome (Morex V3 assembly) (Mascher et al., 2021). Gene-specific overlapping primers (**Supplementary File 1**) were designed using DNAMAN5.0 software. Specific PCR products from genomic DNA and cDNA of E934, *msnsd* and Harrington were sequenced and analyzed using DNAMAN5.0.

### 2.10 RNA preparation and transcriptome sequencing

Immature spikes of *msnsd* and E934 from the glume primordium to the lemma primordium stages, approximately 0.5–1 cm in length, were collected and frozen in liquid nitrogen, with three biological replicates for each sample. Total RNA extraction, library construction and RNA-seq were performed at Beijing Novogene Bioinformatics Technology Co. Ltd, Beijing, China. Clean reads of each sample were mapped to the reference Morex V3 assembly (Mascher et al., 2021). Transcript quantification from RNA-seq data was performed using the Salmon software package (1.9.0). Expression levels of mapped reads were quantified based on transcripts per kilobase of exon per million mapped reads (TPM) (Patro et al., 2017). The Bioconductor package DESeq2 (Love et al., 2014) was used to perform differential expression analysis. Differentially expressed transcripts (DETs) in *msnsd* and E934 were defined as those with |log_2_ fold change (LFC)| ≥ 1.5 and adjusted P values ≤ 0.05. The GO enrichment analysis of DETs was conducted online (http://wheat.cau.edu.cn/TGT/). GO terms with a corrected FDR < 0.05 were considered significantly enriched. The percentage of DETs for each GO term was calculated based on all transcripts in the Morex V3 assembly.

## 3 Results

### 3.1 Phenotype of *msnsd* compared with wild-type E934

**Figure 1:**
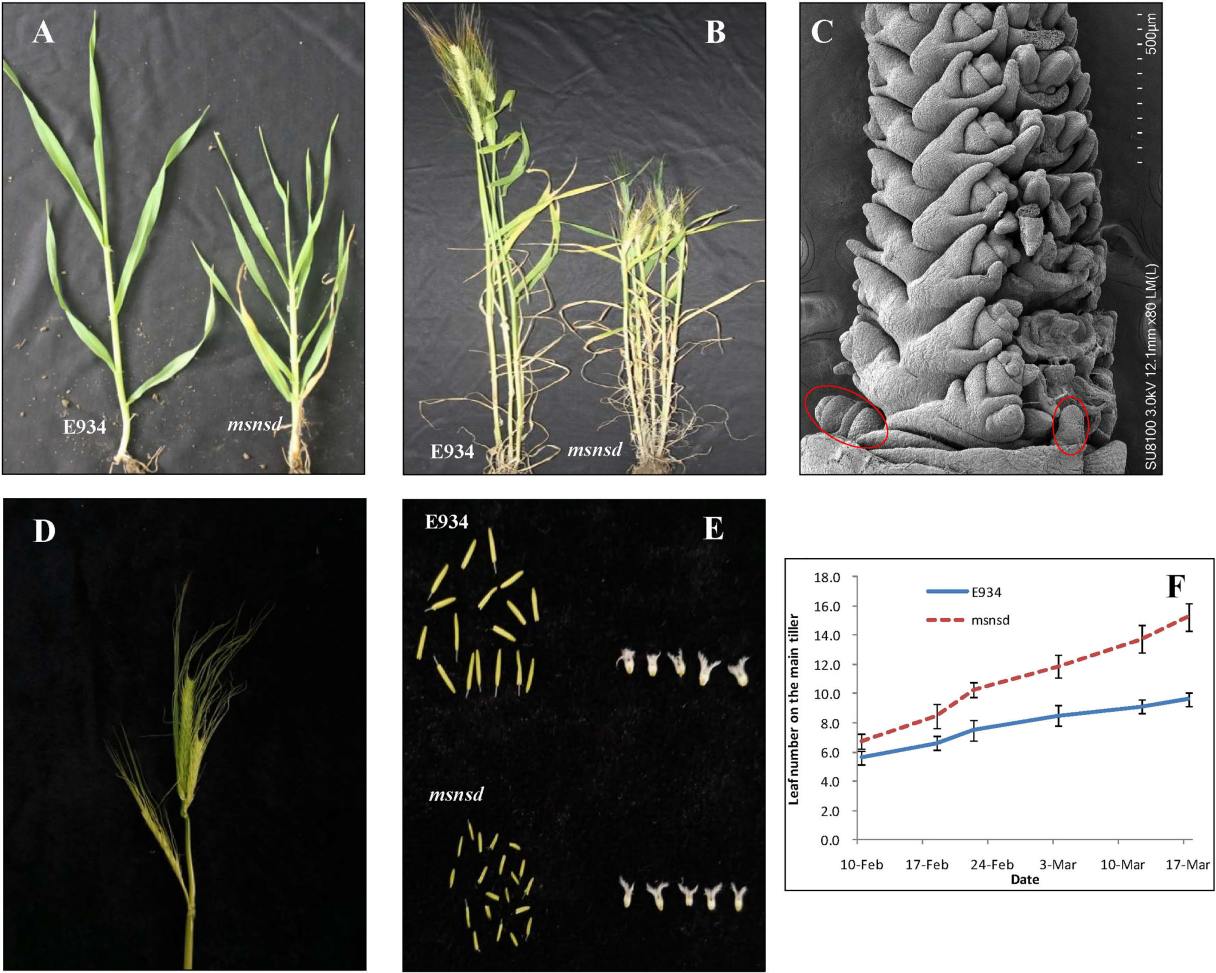
Phenotypes of *msnsd* and E934; **(A)**: E934 and *msnsd* at the jointing stage; **(B)**: Edamai 934 and *msnsd* before maturity; **(C)**: Young inflorescences of *msnsd* (circles indicate an extra inflorescence primordium); **(D)**: Mature spikes of *msnsd;* **(E)**: Stamens and pistils of E934 and *msnsd.* **(F)**: Leaf number on the main tiller of E934 and *msnsd* on different dates.

Compared with E934, the *msnsd* phenotype was characterized as a mutant with multiple stem nodes and spikes per tiller but dwarf (**Figure 1**; **Table 1**), when grown under field and glasshouse conditions. In particular, the *msnsd* mutant had at least four times more stem nodes and leaves than the wild-type (**Figure 1A**) due to a higher rate of leaf initiation (**Figure 1F**). Consequently, the distance between two nodes in *msnsd* decreased significantly relative to E934, such that *msnsd* plants were nearly 40% shorter than wild-type plants (**Figure 1B**; **Table 1**). Apart from the shortened plastochron, *msnsd* had more tillers (**Figure 1B**), but smaller diameter and spike length than E934 (**Table 1**). Moreover, *msnsd* had more than one fertile spike per tiller, located at the base of the main spike or the lower stem nodes, somewhat different from typical branched spikes (**Figure 1D**). These extra mini spikes at the base of the main spike appeared at the beginning of the reproductive stage (**Figures 1C, D**), but all *msnsd* spikes had lower fertility rates than E934. Further analysis showed that *msnsd* had smaller stamens and pistils than E934 (**Figure 1E**). Moreover, all trait alterations in *msnsd* were stable and repeatable, despite being sown one month later or earlier than the normal sowing time in Wuhan, China.

**Table 1:** Agronomic traits of E934 and *msnsd* plants.

	PH (cm)	NSP	LS (cm)	GNS	NN	Length between nodes (bottom to top, cm)									
						1	2	3	4	5	6	7	8	9	10
E934	64.9	4	6.2	29.1	4.1	5.1	8	8.5	12.6	14.7	16.0				
*msnsd*	38.7^**^	3.7	5^*^	25	8.7^**^	1.2^**^	2.7^**^	3.5^**^	3.7^**^	3.7^**^	4^**^	3.5	4.7	5.6	6.1

PH, Plant height; NSP, Number of spikes per plant; LS, Length of the main spike; GNS, Grain number on the main spike; NN, node number on the main tiller.

### 3.2 Microscopic analysis of stem cells in *msnsd* and E934

**Figure 2:**
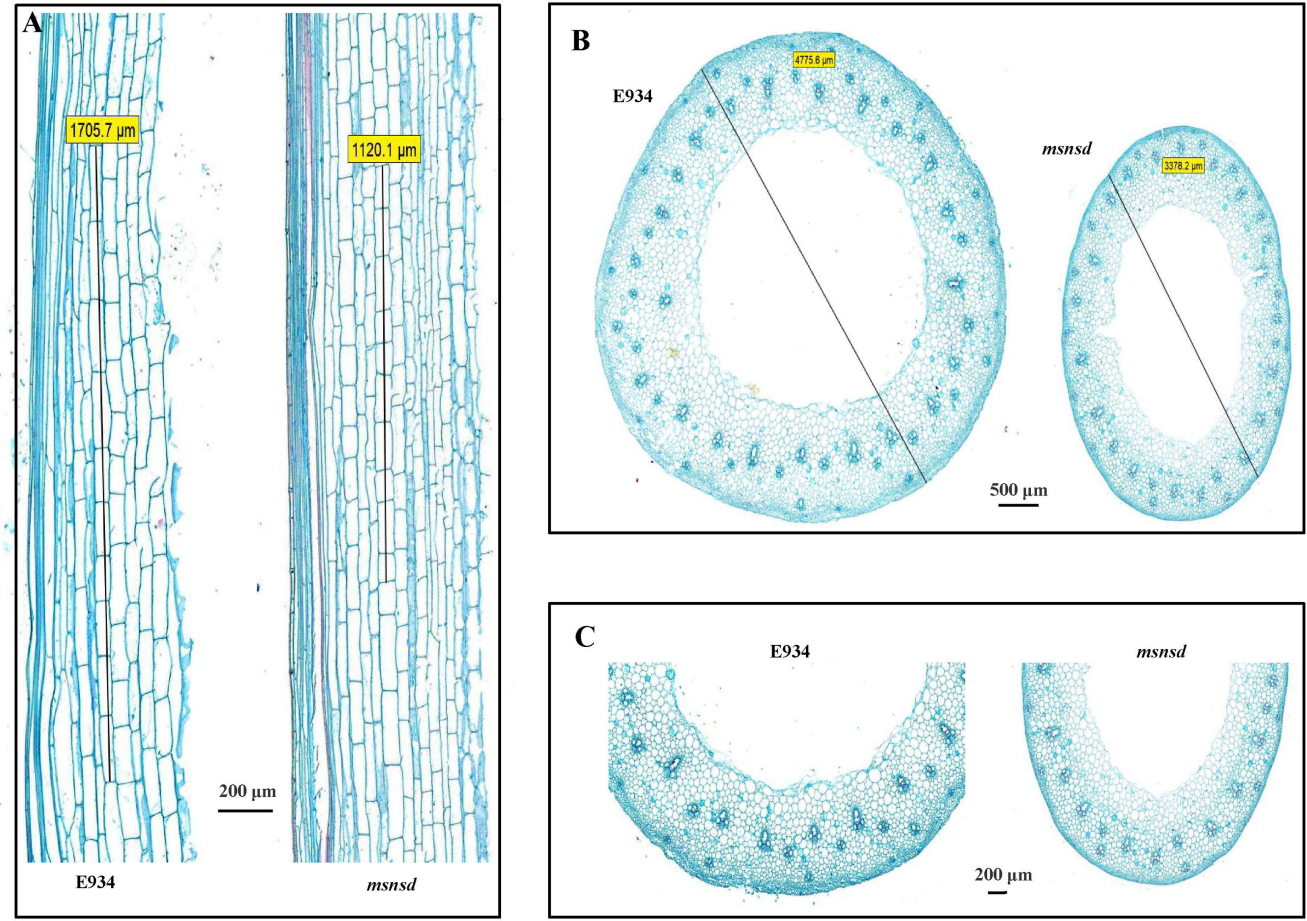
Microscopic analysis of E934 and *msnsd*. **(A)**: the longitudinal culm; **(B)** Transverse sections; **(C)**: Enlarged transverse section. Lines in **(A)** indicate the total length of ten cells. Lines in **(B)** indicate stem diameter.

To determine whether the shorter *msnsd* stems were caused by cell elongation or proliferation changes, we examined the cell morphology of mature stem sections in the center of *msnsd* and E934 plants longitudinally and horizontally under a microscope. As expected, the *msnsd* stems comprised shorter cells than E934, with the mean length of *msnsd* stem cells about three-fourths of those in E934 (**Figure 2A**). While *msnsd* had more tillers than E934, it had much thinner stems than E934 (**Figure 2B**). Microscopic analysis of the transverse section showed that cell size decreased in *msnsd*, especially on the inner side of the stem (**Figure 2C**), as did cell layers (**Figure 2C**).

### 3.3 Analysis of hormone response and quantification of endogenous GA levels

**Figure 3:**
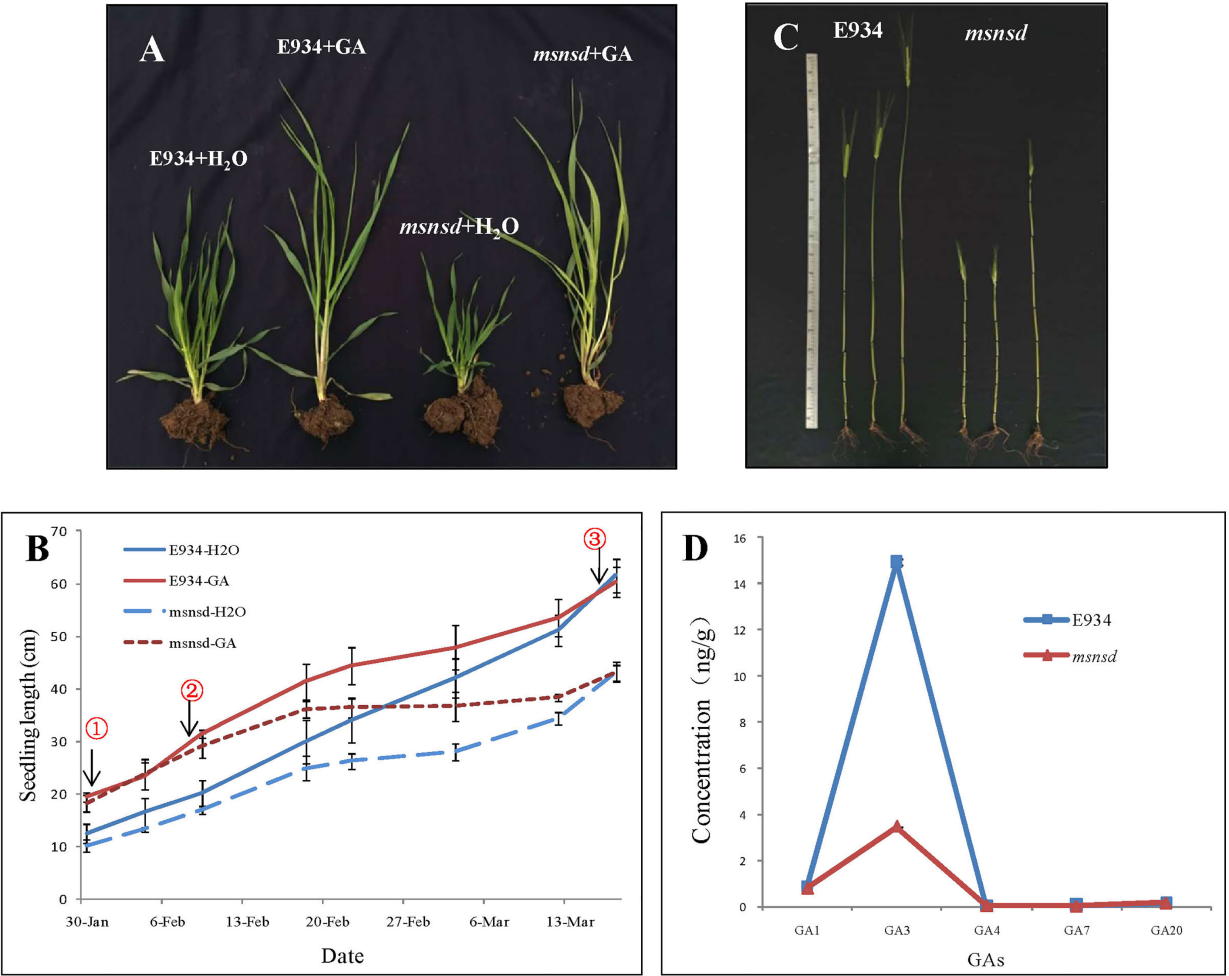
Application and quantification of GA in E934 and *msnsd.* **(A)**: Seedlings of *msnsd* and E934 in the 50 mg/L GA_3_ and control (H_2_O) treatments; **(B)**: Seedling length of *msnsd* and E934 in the 50 mg/L GA_3_ and control (H_2_O) treatments (① Seedling length of *msnsd* and E934 nearly the same with GA_3_ application, ② Growth of *msnsd* plants in the GA_3_ treatment slows, ③ Plant height of *msnsd* and E934 plants nearly the same in the GA3 and water control treatments); **(C)**: Internodes of E934 and *msnsd* in the 50 mg/L GA_3_ and control (H_2_O) treatments (Left to right: control, GA_3_ application at the seeding stage, GA_3_ application at the heading stage); **(D)**: GA concentration in the young apical meristem of *msnsd* and E934 plants.

We investigated the response of E934 and *msnsd* to five different hormones to determine whether the dwarf *msnsd* phenotype was caused by a deficiency in GAs or other phytohormones. At the seedling stage, seven days of GA_3_ application significantly increased E934 and *msnsd* seedling lengths relative to the water treatment (**Figures 3A, B** **)**, with the *msnsd* seedling length almost the same as E934 (**Figures 3A, B** ^①^). However, *msnsd* plants grew slower than E934 plants after about two weeks of GA_3_ application (**Figure 3B** ^②^). Furthermore, the GA and control treatments produced similar internode numbers at maturity for E934 and *msnsd* (**Figure 3C**), contributing to the similar plant heights in both treatments at the same stage (**Figures 3B, C** ^③^). GA_3_ sprayed on E934 and *msnsd* at the heading stage increased internode length and plant height in both of them (**Figure 3C**). However, *msnsd* plants remained shorter than E934 plants in all treatments (**Figure 3C**). Except for plant height, all other investigated traits did not differ between the water and hormone treatments and neither *msnsd* nor E934 plants responded to treatment with other hormones in terms of plant height.

The concentrations of GA_1_, GA_3_, GA_4_, GA_7_ and GA_20_ in the young apical meristem (excluding inflorescence) in *msnsd* and E934 were determined at the booting stage. The GA_3_ concentration significantly decreased in *msnsd* to about one-fifth of that in E934 (**Figure 3D**). However, the concentration of other GAs did not significantly differ between E934 and *msnsd*.

### 3.4 Genetic analysis of the gene controlling multiple stem nodes and spikes but dwarf phenotype of barley

The *msnsd* mutant was crossed with Harrington to characterize the gene underlying the *msnsd* phenotype. All F_1_ plants showed the wild-type phenotype as Harrington, but individuals of the F_2_ population showed segregation between the wild-type and mutant (multiple stem nodes, dwarf and multiple spikes per tiller) phenotypes. The segregation ratio of wild-type individuals to mutants was 340/109, or 3: 1 ratio using a Chi-square test. Moreover, among the 283 F_2:3_ lines derived from wild-type F_2_ individuals, 99 had the homozygous wild-type phenotype and 184 showed segregation of the phenotype within the family. All 89 F_2:3_ families derived from the *msnsd-*type F_2_ individuals showed a homozygous mutant phenotype, suggesting that the multiple nodes and spikes phenotype in *msnsd* followed a simple Mendelian inheritance pattern, with traits behaving in a single and recessive pattern.

### 3.5 Initial mapping of *HvMSNSD* by BSR-seq analysis

Using pooled RNA samples from 40 wild-type and 40 mutant individuals derived from F_2:3_ families and two parents, 1,445 SNPs or Indels were identified as likely associated with the *HvMSNSD* gene, with most (1,409) located on chromosome 5H (**Table 2**; **Supplementary File 2**). Further analysis showed that among these markers, 1,012 were between the 519 Mb and 577 Mb positions based on the Morex V1 assembly, whereas 389 were between the 638 Mb and 668 Mb positions, suggesting that the *HvMSNSD* gene was on chromosome 5H.

**Table 2:** Number and distribution of SNPs and Indels on each chromosome, identified by BSR-Seq.

Chromosome	Position (Mb)	Number of SNPs/Indels
1H	233	2
	538	1
2H	31	1
	151	6
3H	637	3
	698	2
	230	8
5H	519–577	1012
	638–668	389
6H	113	1
	580	5
7H	2	1
	15	18
Total		1428

### 3.6 Sequence of *MND4* and *MND8* in *msnsd* and E934

Studies have shown that *MND4* (HORVU5Hr1G081060) and *MND8* (HORVU5Hr1G118820) are on chromosome 5H. Further analysis revealed that *MND4* and *MND8* are in the intervals between 562934428–562936259 and 653835747–653839974 bp, respectively, coincident with the two intervals of the candidate gene *HvMSNSD* identified by BSR-seq. Therefore, *MND4* and *MND8* were cloned from E934 and *msnsd* to verify whether they caused the *msnsd* phenotype. The results showed that *msnsd* and E934 had the same sequence for the coding region of *MND4* and *MND8*, suggesting that they might not be the causal genes for *msnsd* in the present study.

### 3.7 Validation of candidate gene markers and preliminary mapping of *HvMSNSD*

**Figure 4:**
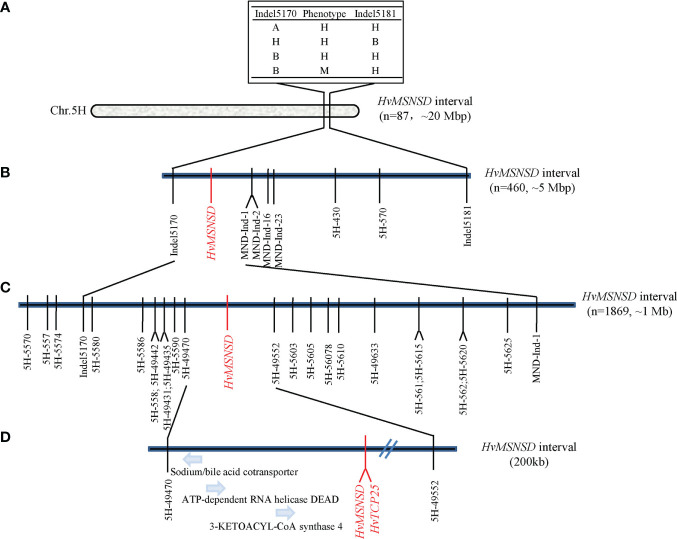
Map-based cloning of *HvMSNSD*; **(A)**: Preliminary mapping of *HvMSNSD*; **(B, C)**: Fine-mapping of *HvMSNSD*; **(D)**: Candidate genes in the target region.

Several markers on chromosome 5H were designed based on the Indels identified in the BSR-seq analysis, with their flanking sequences and other Indel markers around the candidate region obtained from published results. The markers were firstly used to screen Harrington and the *msnsd* mutant, with 14 (**Supplementary File 1**; **Supplementary Sheet 1**) showing polymorphisms between the two parents. The markers were then used to genotype 83 F_2:3_ families derived from 46 wild-type and 37 mutant F_2_ individuals. The results showed that 10 of the 83 families were recombinant families, representing four types of genotypes and phenotypes (**Figure 4A**). According to the phenotypic and genotypic data of the 10 recombinant families, the *HvMSNSD* gene was then mapped to the region between Indel5170 (557, 861, 942 bp) and Indel5181 (578, 321, 468 bp) on chromosome 5H (**Figure 4A**), which excluded *MND8* from further analysis.

### 3.8 Fine-mapping of *HvMSNSD*

Indel5170 and Indel5181 were applied to the F_2_ population comprising 460 individuals to fine-map *HvMSNSD*. Five polymorphic markers—three Indel markers and two SNP markers in the target region—were further developed according to the BSR-seq analysis results, delimiting *HvMSNSD* to the region between Indel5170 and MND-Ind-1, spanning about 5 Mb (**Figure 4B**; **Supplementary File 1**).

Furthermore, 24 polymorphic SNP markers around the target region were developed and applied to 1,869 F_2:3_ and F_3:4_ homozygous mutant progenies derived from the heterozygous lines. Finally, the gene was narrowed to the region between 5H-49470 (559, 135, 754) and 5H-49552 (560, 194, 831), spanning about 1 Mb on chromosome 5H (**Figure 4C**), further indicating that the reported *MND4* gene was not the causal gene for *msnsd* in this study.

The barley reference genome sequence identified 13 genes within the 1 Mb target region of *HvMSNSD*, with only TCP family members reportedly associated with the spikelet morphology of barley. Therefore, this TCP gene was considered the most likely candidate causal gene for *msnsd* and subjected to further cloning and sequence analysis.

### 3.9 Cloning and analysis of *HvTCP25*

Based on the barley reference genome sequence, the TCP gene in the target region is the previously identified *HvTCP25* gene, which was 999 bps without intron with a TCP conserved domain at the N-terminal of an amino acid coding sequence (interval of 65^th^-126^th^ amino acid). The coding regions and the 800 bps upstream regions of *HvTCP25* from *msnsd*, E934 and Harrington were cloned and sequenced, revealing that the coding region was 1005 bps without intron and *msnsd* had a C-T substitution at +663 in the nearly 2000 bps compared to E934 and Harrington, with no change in amino acids (TTC to TTT).

A PARMS marker (**Supplementary File 1**) was developed based on the C-T variation and used for genotyping the F_2_, F_3_ and F_4_ populations comprising 2,329 individuals. Interestingly, the C-T SNP co-segregated with the *msnsd* phenotype in the large population. Consequently, the *MSNSD* interval was delimited to the 200 kb interval between 5H-49470 and 5H-TCP (**Figure 4D**), which also confirmed that the known *MND4* was not the underling gene for *msnsd*. In addition to *HvTCP25*, three other known genes were identified in the 200 kb region, including sodium/bile acid cotransporter, ATP-dependent RNA helicase DEAD and 3-ketoacyl-CoA synthase 4 (**Figure 4D**). However, none of these genes had sequence variations likely associated with the *msnsd* phenotype after their sequencing in E934, Harrington and *msnsd*.

### 3.10 Transcriptional profiling by RNA sequencing

Transcriptome analysis was conducted on 0.5–1 cm young panicles of *msnsd* and E934 to determine the potential target genes of *HvMSNSD* that regulated barley development. A set of 118 DETs were discovered between *msnsd* and E934 (**Supplementary Files 3**, **4**), with 83 and 35 DETs upregulated and downregulated, respectively, in *msnsd* compared with E934. According to the RNA-seq data, *HvTCP25* had a slightly but not significant lower expression level in *msnsd* than E934, but interestingly, the *HvMSNSD* mutation upregulated the recently cloned *MND1* gene (HORVU.MOREX.r3.7HG0742750) more than four-fold, while the other two *MNDs* (*MND4* and *MND8*) were not responsive. Moreover, expression of the other three genes in the target region, including sodium/bile acid cotransporter, ATP-dependent RNA helicase DEAD and 3-ketoacyl-CoA synthase 4, was not changed by the mutation of *HvMSNSD*.

Though *msnsd* was GA-deficient and responded to GA application (GA-responsive), no GA biosynthesis and GA metabolisome related genes was identified to be DETs. Next, the 118 DETs were subjected to GO enrichment analysis to identify pathways likely regulated by *HvMSNSD*. Eleven GO terms were significantly enriched (**Table 3**). Strikingly, three of the six enriched GO terms in Biological Process—”post-embryonic plant morphogenesis”, “mRNA transcription” “ and “response to light stimulus” —were related to three light-dependent short hypocotyl (LSH)-like proteins/genes, which were upregulated in *msnsd* compared with E934. In addition, two TERMINAL FLOWER1 (TFL1) genes belonging to the GO term “negative regulation of flower development” were upregulated in *msnsd* (**Supplementary File 4**). Moreover, some known genes controlling inflorescence development in barley showed differential expression in *msnsd* and E934. For example, the expression of a member of MADS-box gene family (HORVU.MOREX.r3.7HG0664320) increased in *msnsd*, while the expression of BTB/POZ and TAZ domain-containing protein (HORVU.MOREX.r3.2HG0176250) decreased (**Supplementary File 4**).

**Table 3:** Enriched GO terms.

GO term	Description	Ratio in foreground	Ratio in background	FDR	GeneID (HORVU.MOREX.r3.)
Biological_Process					
GO:0090698	post-embryonic plant morphogenesis	3/47	10/18094	0.000169	2HG0181760 6HG0596190 1HG0071800
GO:0009299	mRNA transcription	3/47	12/18094	0.000169	2HG0181760 6HG0596190 1HG0071800
GO:0009061	anaerobic respiration	2/47	5/18094	0.00208	7HG0703280 7HG0703270
GO:0016540	protein autoprocessing	2/47	10/18094	0.00697	2HG0204250 2HG0204260
GO:0009416	response to light stimulus	4/47	159/18094	0.0145	2HG0181760 6HG0596190 1HG0071800 7HG0670450
GO:0009910	negative regulation of flower development	2/47	22/18094	0.0234	4HG0407080 2HG0166090
Molecular_Function					
GO:0004067	asparaginase activity	2/53	6/19365	0.00652	2HG0204250 2HG0204260
GO:0008798	beta-aspartyl-peptidase activity	2/53	8/19365	0.00652	2HG0204250 2HG0204260
Cellular_Component					
GO:0005797	Golgi medial cisterna	3/60	56/19782	0.0184	5HG0486560 7HG0703280 7HG0703270
GO:0099503	secretory vesicle	3/60	103/19782	0.0422	6HG0620630 6HG0611080; 2HG0205530
GO:0048046	apoplast	6/60	513/19782	0.0422	6HG0620630; 7HG0665050 5HG0429970 2HG0166450 6HG0617040 6HG0617020

## 4 Discussion

### 4.1 *msnsd* may be a valuable resource for barley breeding and study

The multiple nodes and dwarf (*mnd*) mutants of barley are a valuable resource for detecting genes that control shoot branching and vegetative versus reproductive growth (Walla et al., 2020), with several *mnd* mutants identified in the last century. *msnsd* is a “multiple stem nodes and spikes and dwarf” mutant of barley identified from the ^60^Co- and EMS-treated E934 variety. The pleiotropic alteration is independent of external conditions and sowing date in *msnsd*, which differs from *mnd*, whose dwarf phenotype could only be observed under field conditions but not in glasshouse (Mascher et al., 2014). In addition, *msnsd* provides a valuable genetic resource for lodging resistance according to its field performance. Spikes per plant and grains per spike are two of the three most important yield components for the grass family, therefore, the increased tillers and multiple spikes per tiller in *msnsd* could be favorable traits for barley breeding. However, the higher rate of infertility of these external spikes due to decrescent reproductive organs decreases the grain number in *msnsd*. While several *mnd* mutants have been identified, only three genes associated with *mnd* have been cloned (Mascher et al., 2014; Walla et al., 2020; Hibara et al., 2021). Therefore, characterizing *msnsd* and its causal gene will be helpful for illustrating barley morphogenesis and breeding barley varieties with lodging resistance and higher yield potential by optimizing the balance between spike number and fertility rate.

### 4.2 Dwarf phenotype of *msnsd* caused by inhibited cell elongation and shortened phyllochron

It is well known that dwarf phenotypes of plants are induced by shortened cells, inhibited cell division, or both. In wheat, peduncle tissues of DD399 comprised shorter cells than the wild-type ND399, which may have reduced its plant height (Wu et al., 2021). Likewise, the length of parenchyma cells significantly decreased in the maize dwarf mutant *dnl2* (Han et al., 2022). In barley, the significantly reduced length of the uppermost internode in the mutant Sheathed Spike 1 (SS1) was attributed mainly to suppressed cell elongation (Pu et al., 2021). However, the reduced plant height of two maize dwarf mutants, *short internode length1* (*sil1*) and *short internode length2* (*sil2*), was caused by decreased cell numbers and longitudinal cell size (Li et al., 2020). In the present study, the cell length of mature stems decreased in *msnsd*, suggesting inhibited cell elongation. Moreover, successive leaves on the main culm of *msnsd* appeared faster than E934, with a shorter time interval from node initiation to maturity. Thus, it was inferred that an inhibited cell elongation and shorter phyllochron of *msnsd* caused shorter internodes and the dwarf phenotype. In addition, the decreased cell size and cell number may contribute to the reduced stem diameter and thickness in *msnsd*.

### 4.3 *msnsd* was gibberellin-deficient but GA-sensitive

GA is a well-known phytohormone that plays a key role in plant growth and development, especially plant height. If exogenous GA application promoted plant growth, the plant was considered GA-sensitive; otherwise, it was called GA-insensitive (Ellis et al., 2004). Many studies have been conducted to study the effect of GA on plant height. The known dwarfing genes *Rht-B1b* and *Rht-D1b* in wheat were considered GA-insensitive dwarfing genes due to their reduced response to GA (Peng et al., 1999), whereas the plant height of wheat *Rht12* dwarf lines increased by more than 50% in the GA_3_ treatment (Chen et al., 2014) and the wheat dwarf mutant DD399 also had significantly longer seedling and coleoptile lengths in the GA treatment than the non-treated control (Wu et al., 2021). Some dwarf mutants in plants, like the dwarf and narrow-leaf mutant *dnl2* in maize, are GA biosynthesis-deficient mutants, with significantly lower GA concentrations than the wild-type mutant (Han et al., 2022). Inhibition of UI parenchyma cell extension in barley *SS1* was possibly related to insufficient amounts of endogenous bioactive gibberellins (Pu et al., 2021). In the present study, GA_3_ application promoted stem elongation in E934 and *msnsd* plants at the vegetative and reproductive stages, suggesting that these plants are GA-sensitive, with *msnsd* mutants deficient in GA biosynthesis rather than GA signal transduction. Further quantification of GAs in apical meristems showed that the GA biosynthesis, particularly GA_3_, was significantly inhibited in *msnsd*. Interestingly, the increase in plant height in *msnsd* slowed after 14 days of GA application at the seedling stage and almost stopped after 28 days, indicating that the GA sprayed on *msnsd* seedlings had been used up after one month. GA application at the heading stage also increased plant height, confirming that the GA amount was insufficient, but external GA application could trigger GA signaling in *msnsd*. The GA_3_ treatment did not change spike performance in *msnsd*; however, the crosstalk between hormones such as auxin, cytokinin, gibberellins and abscisic acid along the spike sections played a decisive role in spike and spikelet development of barley (Youssef and Hansson, 2019), suggesting that the morphology of stem and spike of *msnsd* formed through independent pathways. In barley, GA_3_-treated *vrs2* also exhibited typical symptoms of gibberellin application, such as elongation of stem and spike internodes, but still showed gradients in lateral spikelet fertility along the spike, as observed in the non-treated control plants (Youssef et al., 2017). Genetic studies also confirmed that the rice PLASTOCHRON genes *PLA1* and *PLA2* acted downstream of the GA signal transduction pathway to regulate leaf growth by affecting cellular proliferation (Mimura et al., 2012; Mimura and Itoh, 2014).

### 4.4 *HvTCP25* might be the causal gene for *msnsd*, with pleiotropic effects on stem and spike development

Among the identified barley multiple stem nodes and dwarf mutants in the current study, some mutants had similar spike performance to the *msnsd* mutant, developing more than one spike on each tiller. A genetic analysis showed that two independent but closely linked recessive genes controlled some of the multiple nodes and dwarf mutant phenotypes (Xu and Huang, 1999). However, some previous studies (Li and Li, 1994) and our study showed that a single recessive gene controlled ‘spike variation and multiple nodes and dwarf’ phenotypes, which exerted pleiotropic effects on stem and spike morphology in barley.

To date, three genes responsible for this phenotype in barley (*MND1*, *MND4* and *MND8*) encoded Acyl-CoA N-acetyltransferase-like protein, cytochrome P450 and MATE family transporter protein, respectively. While *MND4* and *MND8* were on chromosome 5H, the mapping and sequencing analysis showed they were not the causal gene for the *msnsd* mutant in this study. Screened by BSR-seq and map-based cloning, the *MSNSD* gene was mapped on chromosome 5H in a 1 Mb interval. Among the 13 high-confidence genes in the 1 Mb target region, only plant-specific *TCP* transcription factor family genes have been reported responsible for branched spike formation in barley. According to published public datasets, 20 TCP genes encoding mature proteins have been identified in barley (Gao et al., 2021). Mutation in the CYC/TB1-type *TCP* on chromosome 5H produced a branched inflorescence in the *bdi1* mutant (Shang et al., 2020), while another member COMPOSITUM 1 specified branch-inhibition in barley (Triticeae) versus branch-formation in non-Triticeae grasses (Poursarebani et al., 2020). Apart from the identity of the spikelet meristem, TCP members also participated in barley seed germination (Seven and Akdemir, 2020) and selection from wild barley (*Hordeum vulgare* ssp. *spontaneum*) to cultivated barley (*Hordeum vulgare* subsp. *vulgare*) (Gao et al., 2021). In the present study, the *TCP* gene in the target region is the known *HvTCP25* gene (Gao et al., 2021), belonging to subgroup class I, as distinguished by the deletion of four amino acids at the N-terminal. Based on the publicly released RNA-seq datasets, *HvTCP25* had a very low expression level across the 21 investigated tissues and developmental stages (Gao et al., 2021), but its function remains unclear. However, the TCP domain of the members of this gene family was quite conserved between barley and its relatives, suggesting that they play similar roles in plant development. In this study, while the multiple spikes per tiller in *msnsd* differed from the branched spike mentioned earlier, re-sequencing of *HvTCP25* in *msnsd*, E934 and Harrington identified a C-T SNP on it. While the C-T variation was a nonsense mutation, it co-segregated with the *msnsd* phenotype in the large mapping population comprising 2,329 progenies, indicating that *HvTCP25* is most likely the causal gene for the *msnsd* phenotype in the current study. However, *HvTCP25*’s precise function and mechanisms in the *msnsd* phenotype remain under investigation.

### 4.5 *HvMSNSD* may regulate barley infloresence development by known genes associated with floral development

Studies on the rice plastochron and barley multiple nodes and dwarf mutants confirmed that the responsible genes independently regulated these traits (Kawakatsu et al., 2006; Hibara et al., 2021). Among the three investigated *MND*s based on our RNA-seq results, the *HvMSNSD* mutation induced *MND1* expression. Further analysis demonstrated that *MND8* was not expressed in young panicles (0.5–1 cm) of E934 or *msnsd*, while *MND4* was expressed in E934 and *msnsd* with no difference between them. These results suggest that *HvMSNSD* regulates barley development through *MND1*. However, both *MND4* and rice *PLA1* encoded plant-specific cytochrome P450 protein family genes, expression of three P450 genes (**Supplementary File 3**) were up-regulated in *msnsd*, implying that they might cause the shorter plastochron in *msnsd*.

While all *mnd* mutants showed a dwarf phenotype, the transcriptomic analysis of *mnd1.a* detected no differential expression of genes related to GA biosynthesis or signaling in GA-signaling pathways (Walla et al., 2020). To be consistent with this, we didn’t detect any differential expression of GA biosynthesis and metabolism related genes either. It probably because that it was young infloresence but not stems was used in transcriptome analysis, which also suggested that *HvMSNSD* participated in development of barley plant height and inflorescences via different pathways.

**Figure 5:**
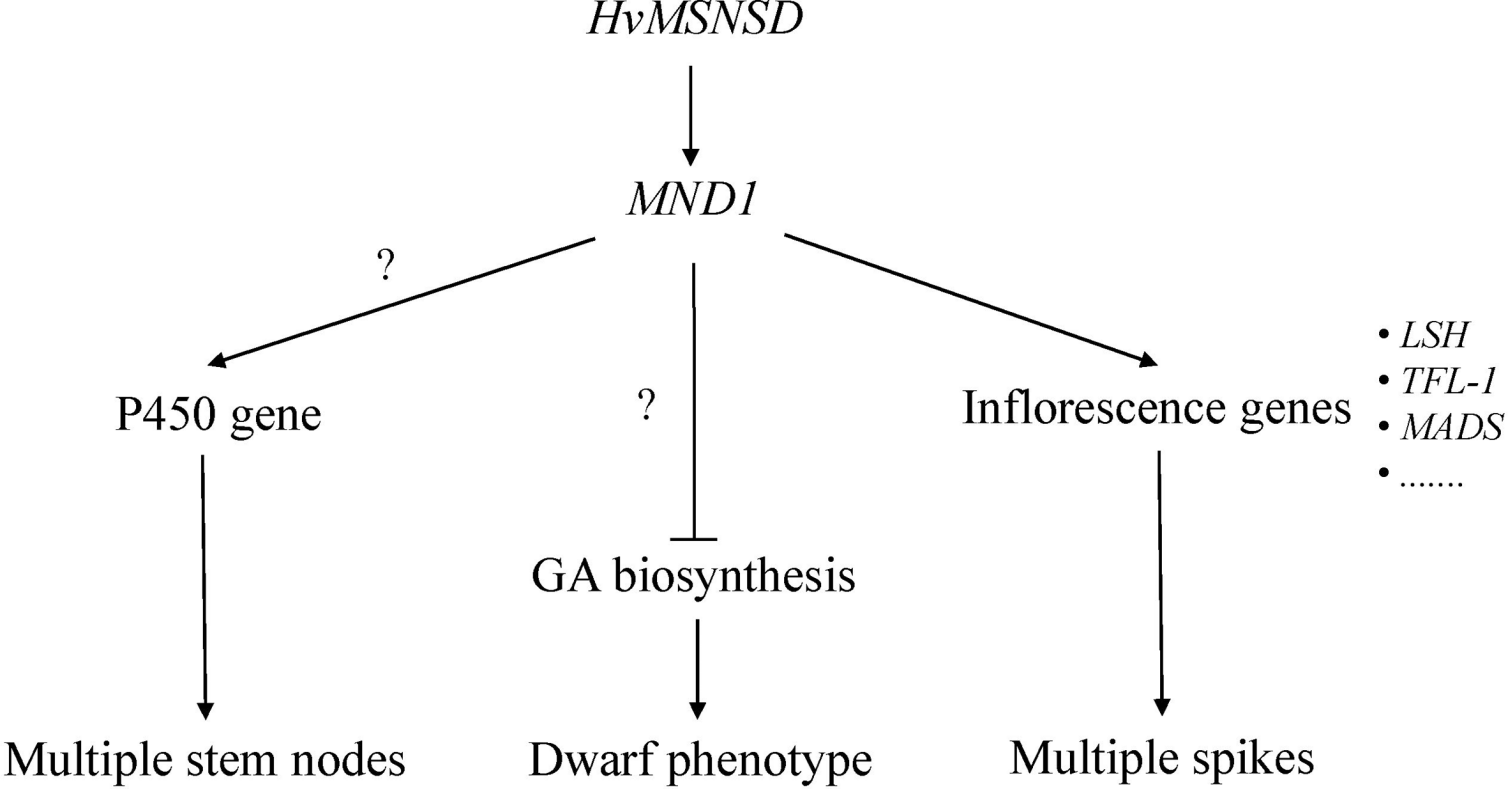
Hypothetical regulatory model for *HvMSNSD* in barley stem and spike development.

For the *mnd1.a* mutant, a core set of DETs comprising 43 transcripts was discovered at all three developmental stages, including developing inflorescences, compared with the wild-type Bowman (Walla et al., 2020). The *msnsd* mutant had more DETs than the wild-type E934. Further analysis identified several overlapped DETs between *msnsd* and *mnd1.a*. For example, *msnsd* and *mnd1.a* upregulated three and four LIGHT-DEPENDENT SHORT HYPOCOTYLS-like (LSH) proteins, respectively, compared with their corresponding wild-type mutants. In addition, both mutants strongly upregulated two TERMINAL FLOWER1-like (TFL-1) genes. However, *mnd1.a* downregulated seven MADS-box genes in inflorescences, with only two upregulated in *msnsd*. All these common genes are known to participate in inflorescence development. Studies on *Arabidopsis* revealed that plants with increased *TCP2* expression displayed the light-dependent short-hypocotyl phenotype (He et al., 2016). The *TFL1* gene from perennial ryegrass involved in floral transition and axillary meristem identity (Jensen et al., 2001) is an essential molecular switch for regulating endosperm cellularization and seed size in Arabidopsis (Zhang et al., 2020a), while the rice *TFL1* gene acted downstream of ethylene in regulating flowering time (Yin et al., 2017). Additionally, studies on Asteraceae proposed that the C class MADS-box TF GAGA1 contributed to stamen development upstream of *GhCYC3*, belonging Mascher et al., 2021 to the TCP protein family (Zhao et al., 2020). Recently, loss-of-function in *HvMADS1* was responsible for developing the branched inflorescence-like structure in barley at high ambient temperatures (Li et al., 2021). Based on the common DETs identified in *msnsd* and *mnd1.a*, *HvMSNSD* and *HvMND1* act as upstream regulators to control inflorescence development in barley through some shared molecular pathways. We proposed a possible pathway for *HvMSNSD* in barley stem and spike morphology development in this study (**Figure 5**).

## 5 Conclusions

A “multiple stem nodes and spikes but dwarf” mutant, *msnsd*, was identified from EMS- and ^60^Co-treated barley variety E934. Using the F_2_ population derived from Harrington and *msnsd*, the causal gene was fine-mapped on chromosome 5H in a 200 kb interval, based on BSR-seq analysis results, with the C-T substitution on exon of *HvTCP25* co-segregating with the *msnsd* phenotype in the population. Transcriptomic analysis showed altered expressions of *MND1* and several genes associated with inflorescence development and GA biosynthesis in *msnsd*. We propose that *HvMSNSD*, probably the known *HvTCP25*, regulates the plastochron and morphology of reproductive organs, likely by coordinating the expression of genes involved in GA biosynthesis and floral development in barley.

## Data availability statement

The data presented in the study are deposited in GEO (Gene Expression Omnibus) on NCBI, accession number is GSE235622.

## Author contributions

DQ conducted all experiments and drafted the manuscript. RL performed gene cloning and expression analysis. FX performed the fieldwork. GD analyzed the genomic sequence. QX, YP, LX, and HC took part in DNA extraction. GG, JD, and CL conceived the experiment and revised the manuscript. All authors contributed to the article and approved the submitted version.

## Supplementary material

Supplementary File 1 List and sequence of primer pairs used.

Supplementary File 2 Distribution and p value of putative *HvMSNSD* associated variations.

Supplementary File 3 Distribution of Differential expressed transcripts.

Supplementary File 4 Differentially expressed transcripts in *msnsd* and E934 plants.
