# Supplementary figures and images for: Characterization of a barley (Hordeum vulgare L.) mutant with multiple stem nodes and spikes and dwarf (msnsd) and fine-mapping of its causal gene
Source: Front Plant Sci. 2023 Jul 6;14:1189743. doi: 10.3389/fpls.2023.1189743 (PMC10359901; doi:10.3389/fpls.2023.1189743)

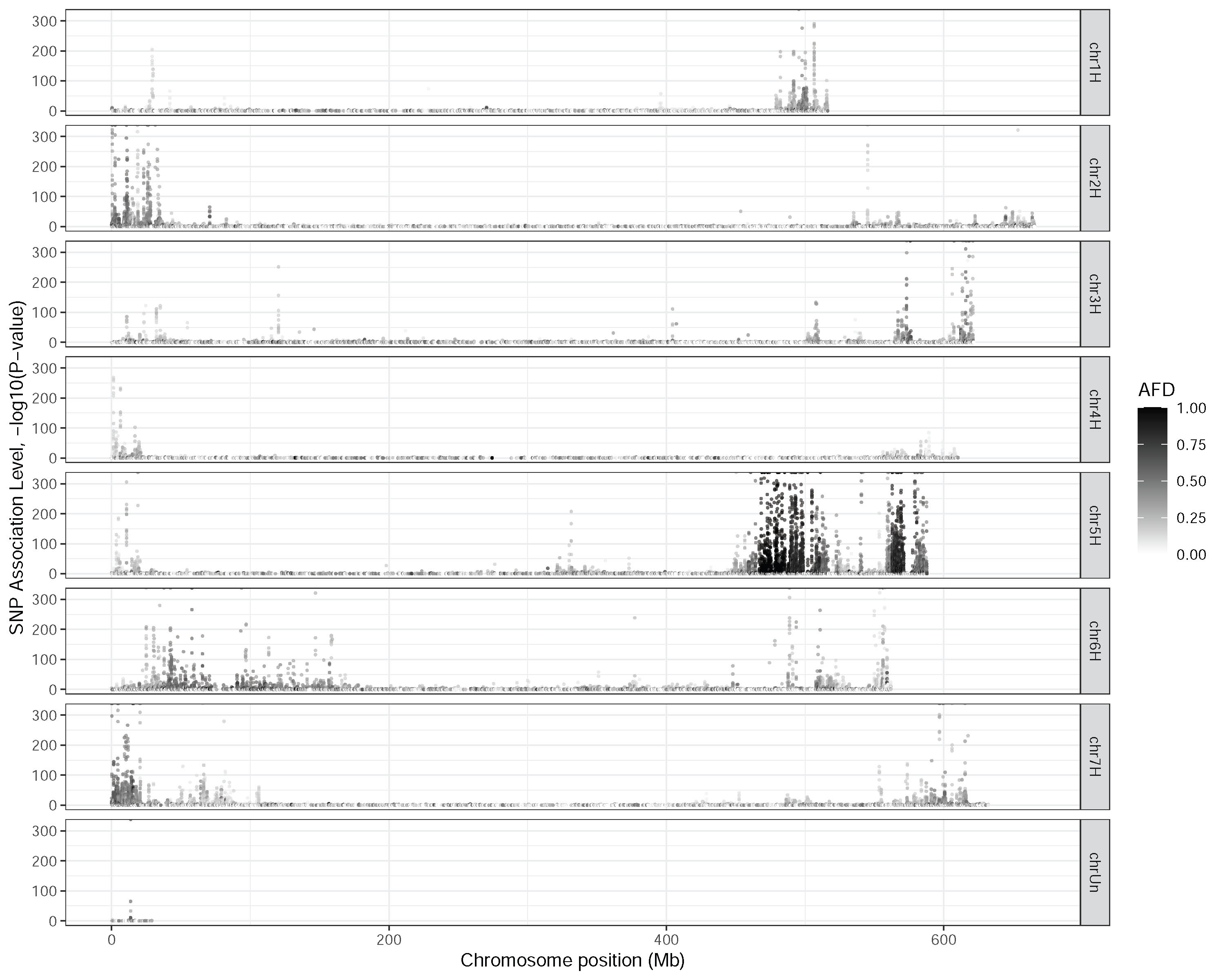

Supplement: Supplementary File 1 — List and sequence of primer pairs used. [file Image_1.jpeg]

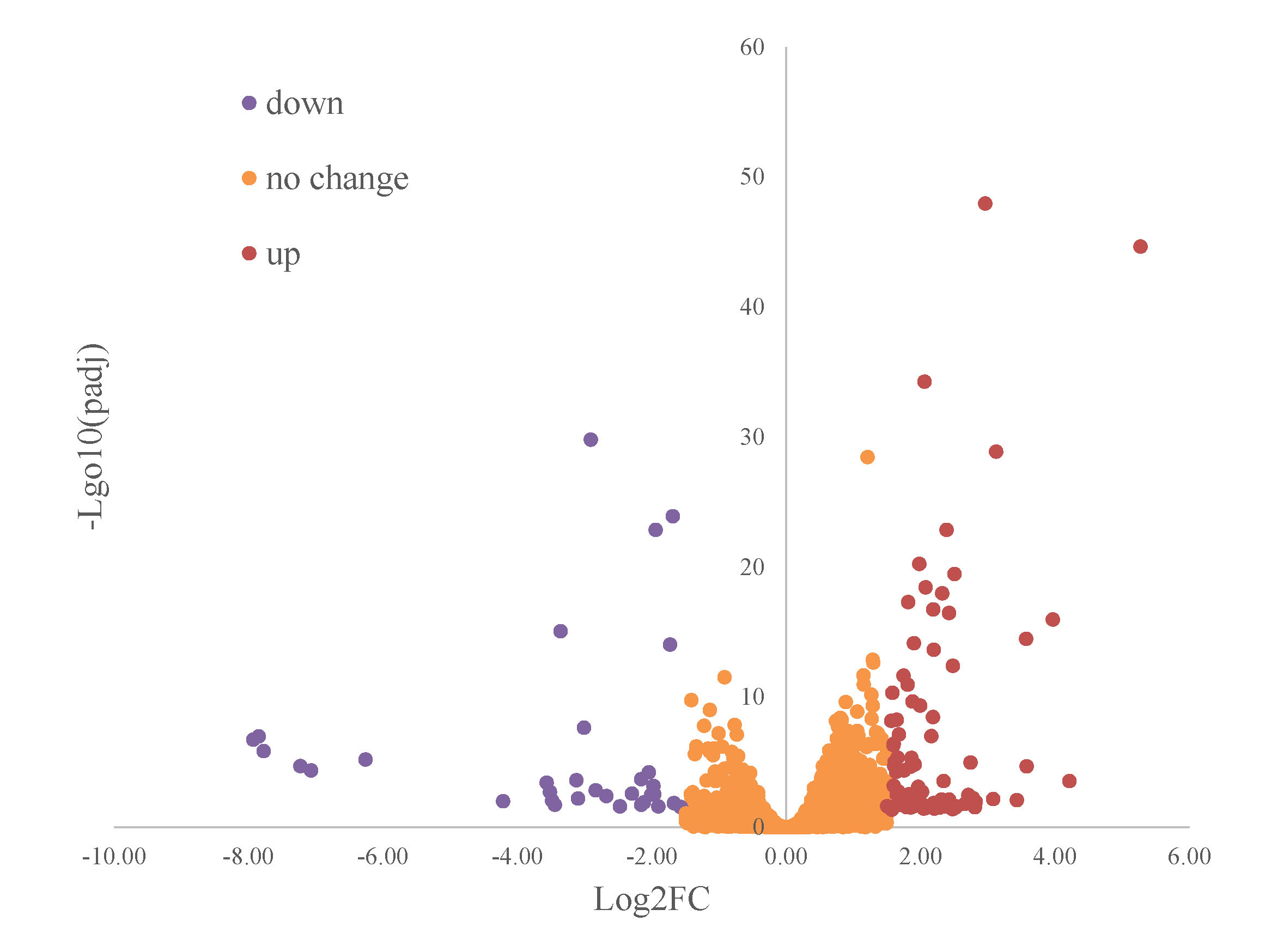

Supplement: Supplementary File 2 — Distribution and p value of putative HvMSNSD associated variations. [file Image_2.jpeg]
